# Bleeding Following Frozen Elephant Trunk Surgery: A Proposed Reporting Dataset and Research Agenda

**DOI:** 10.3390/jcm15218689

**Published:** 2026-11-07

**Authors:** Raluca Elisabeta Staicu, Andrei Raul Manzur, Marciana Ionela Boca, Alina-Ramona Cozlac, Andrea Pop-Crisan, Petru Deutsch, Ana Lascu

**Affiliations:** 1 Clinic of Anaesthesia and Intensive Care, Institute for Cardiovascular Diseases of Timișoara, “Victor Babeș” University of Medicine and Pharmacy Timișoara, Gheorghe Adam Street No. 13A, 300310 Timișoara, Romania; raluca.staicu@umft.ro (R.E.S.); deutsch.petru@umft.ro (P.D.); 2 Department of Doctoral Studies, “Victor Babeș” University of Medicine and Pharmacy Timișoara, Eftimie Murgu Square No. 2, 300041 Timișoara, Romania; 3 Institute for Cardiovascular Diseases of Timișoara, “Victor Babeș” University of Medicine and Pharmacy Timișoara, Gheorghe Adam Street No. 13A, 300310 Timișoara, Romania; marciana.boca@umft.ro (M.I.B.); alina-ramona.cozlac@umft.ro (A.-R.C.); 4 Doctoral School Medicine-Pharmacy, “Victor Babeș” University of Medicine and Pharmacy Timișoara, Eftimie Murgu Square No. 2, 300041 Timișoara, Romania; 5 Advanced Research Center of the Institute for Cardiovascular Diseases, “Victor Babeș” University of Medicine and Pharmacy Timișoara, Eftimie Murgu Square No. 2, 300041 Timișoara, Romania; 6 Department VI Cardiology-Cardiovascular Surgery, “Victor Babeș” University of Medicine and Pharmacy Timișoara, Eftimie Murgu Square No. 2, 300041 Timișoara, Romania; 7 Department of Doctoral Studies, Faculty of Medicine and Pharmacy, University of Oradea, 1 Universității Street, 410087 Oradea, Romania; andrea20_crisan@yahoo.com; 8 Department of Surgery X, “Victor Babeș” University of Medicine and Pharmacy Timișoara, Eftimie Murgu Square No. 2, 300041 Timișoara, Romania; 9 Clinic of Cardiovascular Surgery, Institute for Cardiovascular Diseases of Timișoara, Gheorghe Adam Street No. 13A, 300310 Timișoara, Romania; lascu.ana@umft.ro; 10 Department III Functional Sciences, Pathophysiology, “Victor Babeș” University of Medicine and Pharmacy Timișoara, Eftimie Murgu Square No. 2, 300041 Timișoara, Romania; 11 Centre for Translational Research and Systems Medicine, “Victor Babeș” University of Medicine and Pharmacy Timișoara, Eftimie Murgu Square No. 2, 300041 Timișoara, Romania

**Keywords:** frozen elephant trunk, aortic arch surgery, bleeding, coagulopathy, haemostasis, viscoelastic testing, transfusion, graft-related oozing

## Abstract

Perioperative bleeding after frozen elephant trunk (FET) surgery is reported using heterogeneous endpoints. This structured narrative review examines bleeding sources, measurement and haemostatic management, prioritising recent literature while retaining established landmark definitions. Twelve primary FET clinical publications are tabulated, with technical reports and wider cardiac-surgery evidence providing context. Re-exploration, postoperative drainage, estimated blood loss, transfusion and fabric oozing describe related but distinct phenomena. Established classifications, particularly the Universal Definition of Perioperative Bleeding (UDPB), provide a severity-reporting reference and should not be displaced by a new FET scale. We propose six parallel reporting domains: procedural context; bleeding measurement; haemostatic status; verified source; treatment exposure; and outcomes.. The study-level mapping shows that published FET reports often permit extraction of drainage, re-exploration or treatment exposure, whereas haemostatic status and a directly verified bleeding source are rarely recoverable from published reports. The dataset therefore makes these information gaps explicit and supports prospective evaluation; it does not infer an anatomical source from laboratory findings, drainage or treatment. The proposed reporting dataset is not a validated severity scale or treatment algorithm.

## 1. Introduction

The frozen elephant trunk (FET) technique is an established option for selected patients with complex aortic arch disease extending into the proximal descending thoracic aorta [1,2,3,4,5]. Its wider use has paralleled advances in hybrid prosthesis design, surgical technique, cerebral and distal perfusion, and perioperative care. Nevertheless, FET remains a demanding operation, often involving prolonged cardiopulmonary bypass (CPB), hypothermic circulatory arrest, extensive tissue dissection, large vascular anastomoses, and implantation of a hybrid prosthesis. Together, these features create a haemostatic setting in which systemic coagulopathy and local sources of bleeding may coexist [6,7].

Cardiac surgery already has bleeding classifications: UDPB, the European Multicenter Study on Coronary Artery Bypass Grafting (E-CABG) classification, and the Bleeding Academic Research Consortium (BARC) definitions [8,9,10]. The problem in FET is their inconsistent adoption and incomplete component reporting, not the absence of definitions. FET series instead often emphasise re-exploration, drainage, transfusion or prosthesis-related oozing [1,6,11,12,13,14,15,16]. These measures should be interpreted separately before comparison or aggregation.

Systemic coagulopathy, surgical or anastomotic bleeding, and prosthetic fabric oozing may coexist [7,17,18,19,20,21,22,23]. Their relative contribution cannot be inferred from total drainage or factor administration alone. This matters to anaesthesiologists and surgeons deciding whether a functional haemostatic deficit, a local source, or both require attention. Evolving hybrid devices also differ in construction and implantation technique, although device descriptions do not themselves establish bleeding causality [24,25].

We therefore examine how FET bleeding is measured and managed and propose an operational dataset that complements established severity reporting. Its FET-specific contribution is procedural and prosthetic source attribution, rather than a separate severity scale. Direct FET evidence is distinguished throughout from evidence extrapolated from wider cardiac surgery.

From the perioperative perspective, this distinction changes the question asked of each observation: how much fluid or estimated blood has been lost, what haemostatic function is impaired, which source has been observed, and what response has been delivered? A patient receiving concentrates after prolonged bypass is not thereby shown to have fabric bleeding. Conversely, an anastomotic source found at re-exploration does not exclude an accompanying systemic deficit. Reporting these observations separately makes the clinical reasoning inspectable without claiming that the domains establish causality.

## 2. Methods

### 2.1. Review Design and Literature Search

This study was designed as a structured narrative review. References were managed using Zotero (Digital Scholar, Vienna, VA, USA), and the literature screening process was conducted using Rayyan (Rayyan Systems, Inc., Cambridge, MA, USA). We searched PubMed/MEDLINE, Scopus, and Web of Science for publications addressing perioperative bleeding, coagulopathy, transfusion strategies, coagulation monitoring, and graft-related bleeding in patients undergoing FET surgery. Reference lists of relevant studies and reviews were also screened manually. The literature search was last updated on 23 October 2026.

Search concepts were combined using controlled vocabulary and free-text terms, including “frozen elephant trunk”, “FET”, “aortic arch surgery”, “haemostasis”, “coagulopathy”, “postoperative bleeding”, “blood loss”, “transfusion”, “patient blood management”, “viscoelastic haemostatic assay”, “VHA”, “thromboelastography”, “TEG”, “rotational thromboelastometry”, “ROTEM”, “fibrinogen”, “prothrombin complex concentrate”, “PCC”, “recombinant factor VIIa”, “graft oozing”, and “prosthetic graft bleeding”. Search terms were combined using the Boolean operators AND and OR to identify relevant publications.

Recent evidence published during the six years preceding the final search was prioritised. Older studies were retained for landmark definitions, FET background and mechanistic context. Eligible publications concerned adult FET bleeding, its measurement, monitoring or treatment; contextual arch/dissection and cardiac-surgery evidence was included when relevant. Publications without a relevant haemostatic endpoint or mechanistic contribution were excluded. All authors contributed to the identification, selection and critical appraisal of the literature and to the interpretation of the findings.

The search also included hypothermic circulatory arrest (HCA), deep HCA (DHCA), moderate HCA (MHCA), and viscoelastic testing (VET). Titles, abstracts and relevant full texts informed purposive selection for the narrative synthesis. The documented PubMed strategies and their retrieval counts are provided in Supplementary Table S1; these counts describe the two documented searches rather than the total retrieval across all databases. Supplementary Table S2 identifies the cited publications and their roles. The review did not use an exhaustive systematic screening process.

The review remains narrative: no pooled effect, formal risk-of-bias grading or systematic-review flow is claimed. Reporting was prepared with attention to the six domains of the Scale for the Assessment of Narrative Review Articles (SANRA), a critical appraisal instrument rather than a reporting guideline [26]. Table S2 distinguishes the 12 primary FET clinical publications tabulated from background studies, technical reports, definitions and contextual evidence.

### 2.2. Evidence Synthesis and Development of the Proposal

We extracted study design, population/prosthesis, reported endpoint, collection window, haemostatic assessment, treatment and outcomes. Heterogeneity in definitions, case mix and management precluded meaningful pooling. The reporting domains were developed through interpretative synthesis of these measurement and attribution problems, rather than statistical derivation, formal consensus or external validation. Their proposed time points and verification rules are research specifications, not thresholds established by the included studies.

Evidence was interpreted by design and directness. FET clinical cohorts describe associations and local practice; before-and-after protocols cannot isolate individual components. Technical reports describe feasibility or unusual observations, whereas perfused-graft experiments address permeability under specified conditions. Randomised cardiac/arch studies and professional guidance inform management context but do not provide FET-specific validation. Table S2 makes these roles explicit rather than assigning a numerical evidence grade unsupported by formal appraisal.

## 3. Sources of Bleeding in the FET Setting

### 3.1. Systemic Haemostatic Abnormalities and Procedural Context

FET combines several recognised drivers of acquired perioperative coagulopathy. CPB and haemodilution reduce circulating coagulation factors and platelets; hypothermia impairs platelet function and enzymatic coagulation; extensive tissue injury and contact with artificial surfaces activate coagulation and fibrinolytic pathways; and prolonged operative duration may amplify these disturbances [7,21,22,23,27,28]. Acute aortic dissection may add consumptive and fibrinolytic abnormalities [21,22]. The resulting phenotype may include impaired clot initiation, reduced clot firmness, fibrinogen deficiency, platelet dysfunction, residual heparin effect, and hyperfibrinolysis. These abnormalities are potentially amenable to mechanism-directed haemostatic treatment.

Interpretation should additionally account for urgency, redo surgery, preoperative anaemia, antiplatelet/anticoagulant exposure and concomitant root or valve procedures. Xiao et al. directly studied dual antiplatelet therapy in total arch replacement with FET; the observational findings differed between unmatched and matched analyses and do not justify assuming absence of bleeding risk [29]. Concomitant procedures may prolong bypass and add surgical surfaces. Severe aortic stenosis can involve acquired von Willebrand syndrome, providing a specific reason to record valve pathology rather than assume a uniform haemostatic baseline [30].

Concomitant procedures were recorded in some source studies: Xie’s operative tables distinguish Bentall, valve-sparing and other root procedures [16]. Such information should be extracted alongside bleeding outcomes, not treated as an unmeasured universal characteristic of FET. Recording antithrombotic agent, last administration and emergency presentation is likewise more informative than a single yes/no exposure. Where these covariates or their timing are not available, their absence from the extracted dataset limits adjustment and should not be replaced by assumptions.

### 3.2. Surgical or Anastomotic Bleeding

Not all bleeding after FET is coagulopathic. Surgical sources may persist despite correction of systemic haemostatic abnormalities, particularly in the setting of extensive dissection, friable aortic tissue, complex arch reconstruction, and difficult distal anastomoses. Emergency surgery for acute dissection and greater procedural complexity may further increase this risk. In this setting, escalating blood components or coagulation-factor concentrates without reassessing the operative field may increase treatment exposure without addressing the principal source of bleeding [21,22,31].

### 3.3. Prosthesis-Related Oozing

Clinical reports described E-vita OPEN NEO fabric oozing [17,18]. Tan and Bashir investigated a perfused-graft model, whereas Jakob et al. assessed permeability under specified heparinised in vitro conditions [19,20]. These experimental settings should be described separately rather than treated as interchangeable. Moreover, Jakob’s Essen cohort had 24 h drainage of 1022.1 ± 461.9 mL despite limited in vitro permeability. This illustrates that total drainage is not a clinical measure of fabric permeability; case mix, surgical bleeding, coagulopathy and non-blood fluid can contribute. The Essen and Seoul cohorts reported in that article must also be distinguished [20].

For source documentation, the operative record should identify the prosthesis and configuration, the anatomical site, whether the observation concerns fabric or a suture line, anticoagulation status, temperature and the response to local or systemic measures. A statement that no excessive oozing was observed is different from a protocol measuring permeability. Experimental pressure, blood preparation, equilibration and observation duration should accompany laboratory findings [19,20]. These descriptors help explain apparently discordant reports without implying that differences between experiments establish a clinical ranking of devices.

## 4. Measurement and Existing Bleeding Definitions

**Figure 1:**
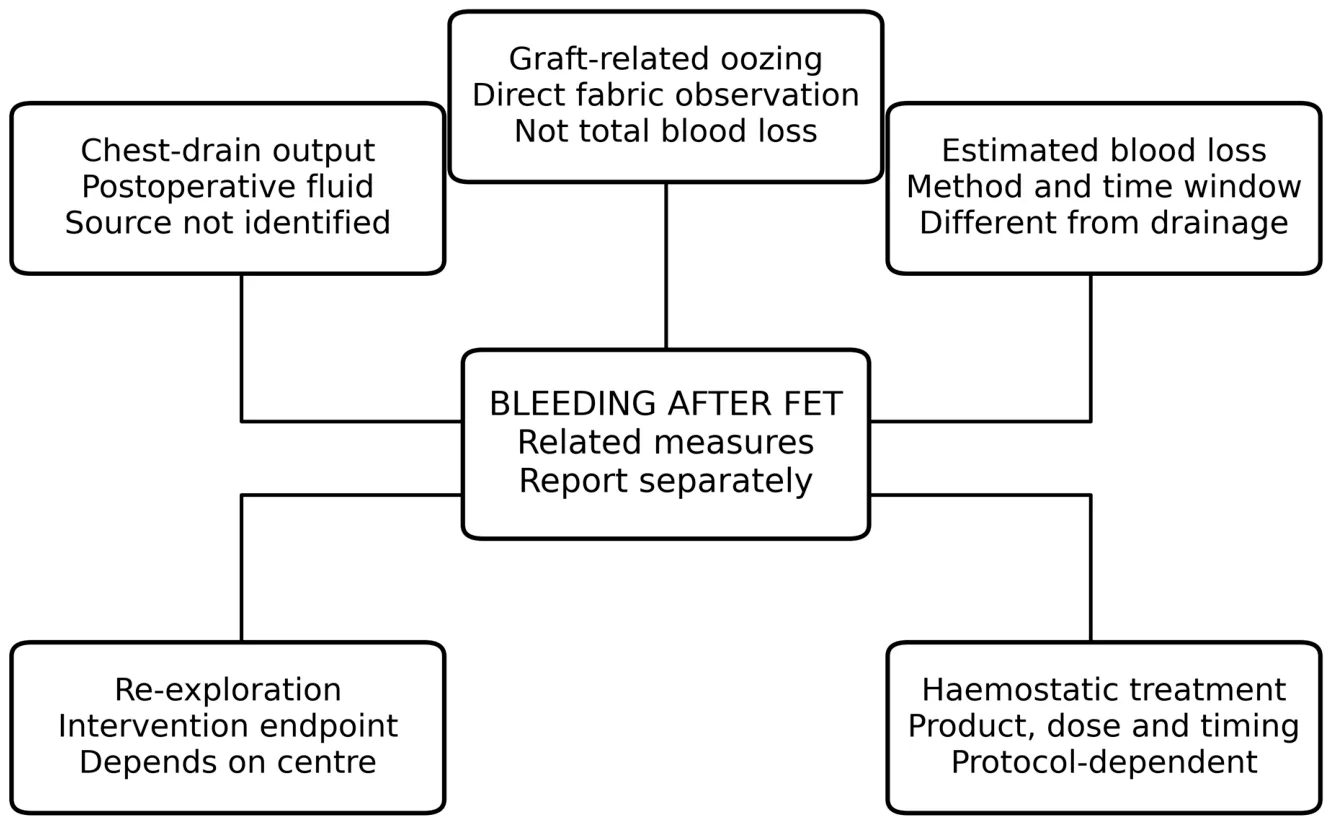
Complementary bleeding endpoints after FET and the limitations of each measurement. Connecting lines indicate association with the central reporting problem, not equivalence between endpoints or a causal pathway. Established severity definitions should be reported alongside these observations. FET, frozen elephant trunk.

UDPB grades adult cardiac-surgery bleeding from class 0 to 4 using chest-tube loss over 12 h, transfusion and consequential interventions [8]. E-CABG emphasises treatment exposure/reoperation, while BARC includes a CABG-related category and was not developed as an FET-specific mechanistic instrument [9,10]. We recommend reporting UDPB with its original rules and individual components, supplemented by the source and procedural domains below. Incomplete retrospective data may prevent reconstruction, but non-reporting does not establish that UDPB is unsuitable for FET. Reasons for non-adoption in individual series cannot be inferred reliably. Figure 1 summarises complementary endpoints and their interpretative limitations.

UDPB itself incorporates treatment and intervention components; it is not a treatment-independent measure of blood loss [8]. Reporting the overall class together with raw drainage, product doses, re-exploration and delayed closure therefore preserves comparability while allowing readers to inspect why the class was assigned. We do not propose altering UDPB thresholds or substituting a 24 h volume for its 12 h component. Additional 24 h measurements are supplementary descriptors. Similarly, a BARC or E-CABG label should be accompanied by its definition and applicable setting rather than treated as interchangeable with another classification.

### 4.1. Re-Exploration

Re-exploration is an important but threshold-dependent event. Rates include approximately 13% in the International E-vita Open Registry and a pooled 7% in the Bashir review [1,6]. The selected contemporary series also report re-exploration/re-thoracotomy, as summarised in Table 1 [11,12,13,15,16,20]. Jakob’s Essen rate was 5.4%; Xie’s unweighted FET group had 15/431 events (3.5%), while its weighted estimate was 3.9% [16,20]. Kozlov reported 0/19 versus 4/26 (15%) for E-vita Open Plus versus MedEng, without a statistically significant difference (*p* = 0.126) [15]. This small comparison cannot establish device effects or institutional decision thresholds.

### 4.2. Drainage and Estimated Blood Loss

Table 1 preserves the reported measurement and collection period rather than equating 24 h drainage, day-of-surgery drainage and postoperative day 1. Postoperative drain output should not be equated with blood loss. As a clinical interpretative consideration, drainage may include non-blood fluid, and fluid balance may be altered in circulatory dysfunction. The cited FET cohorts report drainage outcomes but do not quantify the proportion attributable to non-blood fluid [11,12]. Xie’s blood-loss values require explicit distinction between unweighted and weighted analyses [16]. Demal reported first-24 h loss in primary and redo FET, whereas Kozlov’s postoperative volume requires a separately specified collection interval before harmonisation [14,15]. For future studies, record absolute volumes, body size, the clock origin and the method used to estimate blood volume; any drainage-to-blood-volume ratio remains a drainage descriptor, not a measured fraction of circulating blood lost.

A prospective collection protocol should distinguish blood suctioned before closure, cell-salvage processing and postoperative drain contents, avoiding double counting. Cumulative windows should share an explicit origin and identify periods of drain obstruction, washout or re-exploration that affect interpretation. If output is expressed relative to estimated blood volume, the estimation equation and patient measurements should be retained. This normalisation may aid description across body sizes, but does not remove the non-blood component of drainage or justify labelling the result as a percentage of circulating blood lost.

No validated FET-specific drainage threshold permitting greater postoperative losses on the basis of presumed prosthetic oozing was identified in the reviewed evidence. Haemodynamic stability should be considered alongside the rate and cumulative volume of drainage, haemoglobin trajectory, transfusion requirements and assessment for tamponade or ongoing surgical bleeding [27]. Postoperative drainage alone cannot verify fabric oozing. A suspected prosthetic contribution should be documented as such, rather than used to reclassify high drainage as acceptable or to defer an otherwise indicated surgical assessment.

### 4.3. Treatment Exposure and Graft Observations

Transfusion and factor doses reflect treatment intensity as well as bleeding. Product thresholds, preoperative haemoglobin, haemodilution, test availability, staffing, blood-bank resources and product costs can influence exposure [27,32,33]. These influences are plausible explanations for centre variation, not quantified primary determinants established in FET. Observed fabric oozing should be recorded separately from drainage and re-exploration [13,17,18,19,20]. Broader device coverage is provided by the Thoraflex comparison of Bojko et al., which reports intraoperative transfusion and re-exploration separately; its observational design does not establish equivalence between devices [34].

**Table 1:** Bleeding endpoints and collection periods in representative FET publications.

Study	Population and Prosthesis	Reported Bleeding Endpoint/Result	Definition and Collection Period
Tsagakis registry, 2020 [1]	1165; E-vita Open	Re-exploration 13.0%; 11.7% high-volume versus 16.2% low-volume centres	Postoperative re-exploration for bleeding; a separate fixed-hour collection window is not specified for this endpoint.
Bashir review, 2021 [6]	6313/46 studies; multiple	Pooled reoperation 7%; transfusion aggregated across included series	No single common collection period: study-specific reoperation/transfusion definitions. Pooled estimates are not a single cohort.
Demal, 2021 [14]	85; 54 primary, 31 redo; mainly Thoraflex	Blood loss 781.3 ± 719.0 versus 820.7 ± 498.9 mL	First postoperative 24 h, primary versus redo; an ICU-arrival origin is not established by the extracted endpoint.
Xie, 2021 [16]	633 overall; unweighted TAR + FET *n* = 431	Unweighted FET: blood loss 875.9 ± 617.0 mL; re-exploration 15/431 (3.5%)	Postoperative blood loss/re-exploration; fixed volume-collection interval not specified in outcome table. Weighted estimates are not used here.
Kozlov, 2023 [15]	19 E-vita Open Plus; 26 MedEng	Loss 500 [300–600] versus 600 [500–700] mL; re-sternotomy 0/19 versus 4/26 (15%), *p* = 0.126	Postoperative blood loss; fixed collection window not specified in the source report. Re-sternotomy reported as an in-hospital outcome.
El-Sayed Ahmad, 2023 [11]	22; E-vita OPEN NEO	Drainage 569 [338–910] mL; re-thoracotomy 1/22 (4.5%)	Drain output and transfusion within 24 h. Exact time origin is not explicit in the endpoint label; re-thoracotomy is separate.
Jakob, 2023 [20]	56 Essen patients; E-vita OPEN NEO	Drainage 1022.1 ± 461.9 mL; reoperation 3/56 (5.4%)	24 h clinical drainage in Essen, not an in vitro permeability measure. Separate Seoul cohort *n* = 139 is not pooled into this row.
Kim, 2024 [12]	167; E-vita OPEN NEO	24 h drainage 669 [543–956] mL; day of surgery 500 [370–710]; POD1 360 [280–533]; redo sternotomy 3.0%	24 h, day of surgery and POD1 are distinct reported windows; do not sum them as non-overlapping intervals.
Tsagakis NEOS, 2024 [13]	99 treated; E-vita OPEN NEO	Re-exploration 5/99 (5.1%); graft-material bleeding separately reported	Re-exploration within 24 h after the final suture; distinct graft-related endpoint.
Xiao, 2022 [29]	466; 48 DAPT/418 non-DAPT; 45 matched pairs	Bleeding-related composite; drainage ≥1500 mL/12 h component; matched analyses separate	Composite: reoperation, ≥1500 mL in first postoperative 12 h, ≥10 RBC units or rFVIIa. Do not equate a composite event with drainage alone.
Bojko, 2025 [34]	Matched: 83 Thoraflex/166 traditional FET	Re-exploration 6/83 (7.2%) versus 13/166 (7.8%); *p* = 1.000	Transfusions explicitly intraoperative; re-exploration a postoperative outcome without a fixed-hour limit stated in the source report.

Values are mean ± standard deviation, median [interquartile range], or count (%), as reported. Timing labels are preserved; a clock origin not established from the reported endpoint is not imputed. DAPT, dual antiplatelet therapy; FET, frozen elephant trunk; POD1, postoperative day 1; RBC, red blood cell; rFVIIa, recombinant activated factor VII; TAR, total arch replacement.

## 5. Haemostatic Assessment

### 5.1. Conventional Coagulation Tests and Clinical Assessment

Clinical assessment and conventional tests, including the full blood count, prothrombin time/international normalised ratio (PT/INR), activated partial thromboplastin time (aPTT) and fibrinogen, should be interpreted with temperature, ionised calcium, acid–base status and anticoagulation history [7,27]. Neither conventional nor viscoelastic abnormalities alone identify an anatomical bleeding source; standard assays also do not fully assess antiplatelet effects or von Willebrand factor abnormalities. In a closed chest, source attribution remains provisional until direct verification.

### 5.2. Viscoelastic Haemostatic Assays

Rotational thromboelastometry (ROTEM) and thromboelastography (TEG) provide whole-blood functional information on clot initiation, clot firmness and fibrinolysis [7]. These findings complement clinical assessment and conventional laboratory tests, but do not independently establish the anatomical source of bleeding.

Monaco et al. compared 19 conventional-protocol and 21 ROTEM-era elective FET patients. The published reduction in overall allogeneic blood products was 40%; plasma and platelet exposure decreased, without significant differences in red-cell transfusion, blood loss, reoperation or mortality [32]. Because monitoring changed together with the replacement strategy, including greater use of factor concentrates, the test’s independent effect cannot be isolated. The principal limitation is this confounded before-and-after design, rather than sample size alone. Table 2 distinguishes direct FET management evidence from contextual guidance.

Wider cardiac-surgery trials and guidelines support test-guided haemostatic management, but their trigger values cannot be assumed to have prospective FET-specific validation [27,33,35,36,37]. We identified no prospectively validated FET-specific trigger algorithm in the sources reviewed; the recent FET anaesthetic review is also contextual rather than validation evidence [38].

**Table 2:** Design and interpretation of selected haemostatic management evidence.

Study	Design and Setting	Management/Outcome Information	Interpretation
Monaco [32]	40 elective FET; retrospective before/after	Conventional *n* = 19 versus ROTEM-era *n* = 21; reported 40% lower overall allogeneic products	Bundled protocol association; not the independent causal effect of the test.
Doll [39]	51 FET; retrospective single centre	Transfusion, PCC, fibrinogen and selected rescue agents	Describes institutional treatment exposure; no universal trigger validation.
Ho [17]	Case-based FET technical report	TEG assessment and graft-priming experience	Low-level technical evidence, not comparative proof for routine priming.
El-Sayed Ahmad [11]	22 FET; multicentre cohort	Within 24 h: RBC 7.5 ± 6.5 U; FFP 6.1 ± 5.4 U; platelets 2.5 ± 2.1 U; fibrinogen 2.9 ± 1.9 g; PCC 2484 ± 2104 IU	Treatment burden; do not infer anatomical source from doses.
Jakob [20]	Essen *n* = 56 plus separate Seoul cohort and in vitro tests	Essen: fibrinogen 8.2 ± 4.6 g and PCC 3803.5 ± 2605.3 IU; clinical and experimental outputs separate	Distinct denominators/settings; potential cohort overlap must be considered.
NEOS [13]	99 treated; prospective multicentre interim report	Re-exploration and graft-material bleeding recorded separately	Source-related reporting is useful; not a haemostatic-strategy trial.
Guan [40]	84 acute dissection/arch patients; prospective observational	Low fibrinogen <1.5 g/L; concentrate *n* = 54 versus *n* = 30; drainage at 24/48 h	Contextual arch/MHCA evidence; non-random allocation limits inference.
Vlot [41]	Arch/DHCA randomised trial; *n* = 20; stopped early	Fibrinogen *n* = 10 versus placebo *n* = 10; no demonstrated reduction in loss/transfusion	Contextual arch evidence; underpowered clinical comparison.

DHCA/MHCA, deep/moderate hypothermic circulatory arrest; FET, frozen elephant trunk; FFP, fresh frozen plasma; IU, international units; PCC, prothrombin complex concentrate; RBC, red blood cell; ROTEM, rotational thromboelastometry; TEG, thromboelastography; U, units. Wider cardiac-surgery guidance is contextual [27,33]; the FET anaesthetic review [38] is not a prospective validation study.

## 6. Perioperative Haemostatic Management and Reporting

Preventive and supportive care must be distinguished from rescue factor administration. Tranexamic acid (TXA), red-cell salvage and appropriate heparin monitoring/reversal are established elements of cardiac-surgery blood conservation [33]. In FET, arrest temperature serves organ protection; haemostatic assessment after bypass should document rewarming and the actual core temperature rather than prescribe one arrest-temperature target for all patients. Residual heparin, excessive protamine, hypocalcaemia and acid–base abnormalities require consideration alongside bleeding and laboratory results [27].

The temporal sequence should be documented: assessment during rewarming; evaluation after protamine; deficit-directed replacement with reassessment; and ongoing surgical review. Low fibrinogen should be corrected before considering prothrombin complex concentrate (PCC) for coagulopathic bleeding, because hypofibrinogenaemia itself prolongs clot formation [27]. Platelets, plasma or concentrates should address the documented deficit and clinical bleeding, not an isolated number. Factor XIII (FXIII) testing may be relevant in selected unexplained cases, but routine supplementation with normal concentrations is unsupported. Suspected von Willebrand dysfunction requires specific assessment rather than empirical universal replacement [27,30]. This is a contextual sequence, not a validated FET dosing algorithm.

A verified anastomotic source requires surgical haemostasis; persistent bleeding warrants renewed assessment even when tests improve. Directly observed graft oozing requires device-aware inspection and differentiation from suture-hole bleeding. Local techniques and graft priming have been described, but the technical reports and experimental concerns do not establish routine BioGlue priming as a general solution [17,18,19,20]. Systemic correction and source control may proceed concurrently; urgent surgical assessment should not await completion of a rigid factor sequence. The dose, timing, indication and result preceding each intervention should be recorded.

The evidence for fibrinogen in arch surgery also requires restraint. A prospective cohort under moderate HCA reported favourable associations in patients with low fibrinogen, whereas a prematurely terminated randomised trial under deep HCA did not demonstrate reduced blood loss or transfusion [40,41]. These are contextual arch-surgery data and do not independently validate a universal FET strategy.

## 7. Operational Reporting Domains

**Figure 2:**
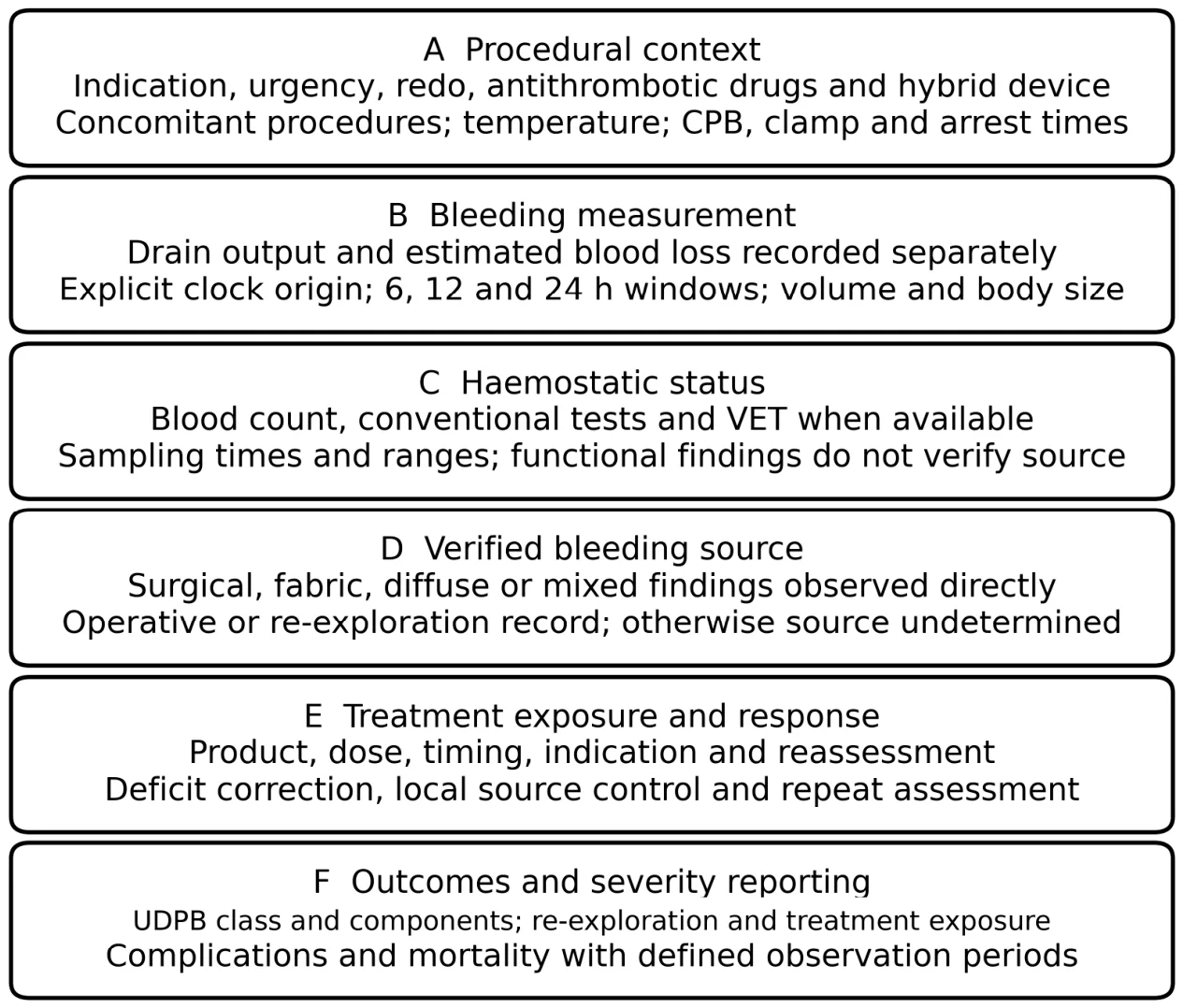
Six parallel domains for FET bleeding reporting. The domains are complementary collection fields rather than consecutive diagnostic or treatment stages. Operational definitions are provided in Table 3. FET, frozen elephant trunk; CPB, cardiopulmonary bypass; VET, viscoelastic testing; UDPB, Universal Definition of Perioperative Bleeding.

The proposal comprises six parallel domains, not a linear sequence. Figure 2 summarises their relationship to established severity reporting; Table 3 supplies operational fields, verification rules and proposed collection points. Timing is standardised to chest closure for future drainage reporting, while the time of intensive-care arrival is retained separately. The source field allows mixed findings and an undetermined category. The same patient may have a verified local source and a functional deficit without either explaining all observed loss.

The study-level map in Supplementary Table S3 applies the reporting domains descriptively to the clinical publications in Table 1 and Table 2. It shows that measurement and treatment exposure are more often extractable than haemostatic status or a directly verified bleeding source. A published drainage value or treatment total does not permit retrospective assignment of an unobserved source. The map identifies what can be extracted rather than treating absent detail as a normal result or reconstructing patient-level UDPB grades from cohort averages. Prospective testing should assess interobserver agreement, data completeness and added explanatory value beyond UDPB.

For example, a report providing a 24 h drainage volume and aggregate transfusion totals can populate measurement and exposure fields only partially if their clock origins differ. It cannot establish the source or recover serial test results. In contrast, an intraoperative note explicitly describing fabric oozing can populate the source field, but does not quantify total postoperative blood loss. The purpose of the retrospective map is to expose these information boundaries. Missing verification remains missing, rather than becoming a negative observation or evidence against a source.

## 8. Discussion

### 8.1. Heterogeneity Is the Central Problem

The useful FET-specific addition is the joint recording of prosthesis, arch/dissection context and directly verified fabric oozing alongside established severity components. It does not follow that six reporting domains are unique to FET; most apply to other complex cardiac operations. Their purpose here is to make explicit the FET variables that otherwise remain obscured when drainage, re-exploration and treatment are presented as interchangeable “bleeding” outcomes.

### 8.2. Bleeding Severity Is Not the Same as Treatment Intensity

Unequal access to VET platforms, trained personnel and rapid laboratory support, together with product availability and costs, may modify treatment exposure at similar measured bleeding. Monaco reported lower local transfusion costs after the bundled protocol change, but this before-and-after result is not a transferable economic effect estimate [32]. Future multicentre work should record these resource characteristics alongside treatment thresholds; comparisons should not interpret lower factor or plasma use automatically as lower biological bleeding severity.

Treatment studies should also retain safety outcomes and their observation periods. Reduced transfusion alone does not establish benefit if re-exploration, thrombotic events or organ dysfunction are incompletely captured. Conversely, an unchanged mortality rate in a small cohort cannot demonstrate safety equivalence. Recording these outcomes separately allows later studies to evaluate the balance between source control, correction of functional deficits and treatment exposure, rather than selecting one favourable endpoint as a summary of the entire postoperative course.

### 8.3. VHA Is Promising, but FET-Specific Validation Remains Limited

Viscoelastic haemostatic assays (VHAs) provide functional information that can support targeted haemostatic treatment. In the Monaco study, however, monitoring was introduced alongside changes in the replacement protocol, so the observed reduction in allogeneic products cannot be attributed to ROTEM alone [32]. Broader cardiac-surgery evidence supports test-guided management, but does not establish the performance of particular triggers in FET [27,35,36,37]. The implication is to evaluate VHAs within a defined care pathway, recording the preceding deficit, treatment and reassessment. Future prospective studies should distinguish monitoring effects from protocol effects and assess bleeding, treatment exposure and safety outcomes together.

### 8.4. Prosthesis-Related Oozing Should Be Reported as a Distinct Phenotype

Directly observed fabric oozing should remain a separate source descriptor [13,17,18,19,20]. Neither drainage volume nor treatment intensity establishes this phenotype; unverified sources remain undetermined.

### 8.5. Future Research Implications

Future validation should first test whether different observers assign the same verified-source category from operative records, including mixed and undetermined categories. It should then assess whether the additional fields explain variation in drainage or exposure after accounting for case mix and an established severity classification. Demonstrating that the dataset can be completed is not equivalent to demonstrating prognostic utility, treatment benefit or fair device comparison. A prospective multicentre design with prespecified collection procedures is needed to distinguish these separate validation questions.

### 8.6. Limitations

Limitations include purposive narrative selection, no formal risk-of-bias appraisal, heterogeneous endpoint definitions and incomplete source verification. Much direct device evidence concerns the E-vita family; adding non-E-vita studies improves breadth but does not remove selection or confounding [14,15,16,34]. Clinical series and their subsequent summaries may overlap and are not independent observations. Experimental permeability, technical reports and uncontrolled cohorts support different claims and should not be assigned equal evidential weight. The proposed domains and collection times need feasibility testing and prospective validation before claims of improved outcomes or device comparability are made.

## 9. Conclusions

FET studies should use an established cardiac-surgery severity definition, report its components and collection windows, and add procedural context and verified bleeding-source information. Graft-related oozing should be documented when directly observed; postoperative drainage alone cannot establish it. The proposed parallel dataset separates these observations from haemostatic treatment exposure and provides an operational basis for prospective evaluation, without introducing another severity scale or claiming a validated therapeutic algorithm.

## Figures and Tables

**Table 3:** Proposed operational dataset for FET bleeding research.

Domain	Operational Fields and Proposed Rule	FET Application
A Context	Indication; urgency; redo; antithrombotic drug/last dose; haemoglobin; valve/root and other concomitant procedures; device/configuration; nadir temperature/site; CPB, clamp and arrest times.	Acute dissection, extensive arch reconstruction, hybrid-device context.
B Measurement	Separate intraoperative estimated blood loss and postoperative drainage. Proposed cumulative 0–6, 0–12 and 0–24 h from chest closure; also record ICU arrival. Report mL, mL/kg and collection method. If normalised to estimated blood volume, retain equation and inputs.	Keep UDPB’s original 12 h component; additional windows do not replace it. Drainage is not blood loss.
C Haemostatic status	Proposed baseline, after protamine, ICU arrival and bleeding/reevaluation samples. Full blood count, PT/INR, aPTT, fibrinogen, VET if available; exact time, values, units and local reference intervals.	Functional abnormalities during/after CPB and rewarming; not anatomical-source proof.
D Verified source	Direct operative observation: anastomotic/surgical, fabric oozing, diffuse or mixed. At re-exploration: surgical, diffuse or both; document site. Without direct verification: undetermined. Keep suspected and verified findings separate.	Fabric versus suture-hole bleeding; do not infer either from total drainage.
E Treatment	Record product/agent, dose, units, time, indication, preceding test and reassessment; cumulative exposure in the same windows as drainage. Separately record TXA, heparin/protamine, cell salvage and source-control procedures.	Deficit-directed correction and local source control are distinct, sometimes concurrent responses.
F Outcomes	UDPB class plus individual components and time windows; re-exploration/time/source; delayed closure; transfusion; thrombotic events; organ dysfunction; mortality and follow-up period.	Permit comparison without claiming source, causality or device equivalence from a composite alone.

These are proposed research collection fields, not validated diagnostic or treatment thresholds. aPTT, activated partial thromboplastin time; CPB, cardiopulmonary bypass; FET, frozen elephant trunk; ICU, intensive care unit; INR, international normalised ratio; PT, prothrombin time; TXA, tranexamic acid; UDPB, Universal Definition of Perioperative Bleeding; VET, viscoelastic testing.

## Data Availability

No new data were created or analysed in this study. Data sharing is not applicable to this article.

## Supplementary Materials

The following supporting information can be downloaded at https://www.mdpi.com/article/10.3390/jcm15218689/s1: Table S1, Documented PubMed search strategies; Table S2, Cited evidence and its role in the narrative synthesis; Table S3, Study-level reporting map for the clinical publications in Table 1 and Table 2 of the revised manuscript. References [1,2,3,4,5,6,7,8,9,10,11,12,13,14,15,16,17,18,19,20,21,22,23,24,25,26,27,28,29,30,31,32,33,34,35,36,37,38,39,40,41] are cited in the Supplementary Materials.
