# Supplementary material for: Bleeding Following Frozen Elephant Trunk Surgery: A Proposed Reporting Dataset and Research Agenda
Source: J Clin Med. 2026 Nov 7;15(21):8689. doi: 10.3390/jcm15218689 (PMC13764511; doi:10.3390/jcm15218689)
Supplement: Supplementary file 1 [file jcm-15-08689-s001.zip › jcm-4637756-supplementary.pdf]

Supplementary Search Strategies and Evidence Inventory

Manuscript jcm-4637756

These materials accompany the revised narrative review. Reference numbers correspond to the revised manuscript. Search yields are distinguished from publications selected for contextual discussion and from primary FET clinical publications tabulated in the main text.

Table S1 Documented PubMed search strategies

A Frozen elephant trunk and haemostasis

Database: PubMed. Run date: 23 October 2026. Publication-date filter: 23 October 2020–23 October 2026. Retrieved records: 105.

```
("frozen elephant trunk"[Title/Abstract]) AND (bleed*[Title/Abstract] OR haemostas*[Title/Abstract] OR
hemostas*[Title/Abstract] OR coagul*[Title/Abstract] OR transfus*[Title/Abstract] OR ooz*[Title/Abstract] OR
viscoelastic[Title/Abstract] OR thromboelast*[Title/Abstract] OR ROTEM[Title/Abstract] OR TEG[Title/Abstract] OR
VET[Title/Abstract]) AND ("2020/10/23"[Date - Publication] : "2026/10/23"[Date - Publication])
```

B Aortic arch circulatory arrest and haemostasis

Database: PubMed. Run date: 23 October 2026. Publication-date filter: 23 October 2020–23 October 2026. Retrieved records: 42.

```
("aortic arch"[Title/Abstract] OR ("frozen elephant trunk"[Title/Abstract])) AND ("hypothermic circulatory
arrest"[Title/Abstract] OR HCA[Title/Abstract] OR DHCA[Title/Abstract] OR MHCA[Title/Abstract]) AND
(bleed*[Title/Abstract] OR haemostas*[Title/Abstract] OR hemostas*[Title/Abstract] OR coagul*[Title/Abstract] OR
transfus*[Title/Abstract] OR ooz*[Title/Abstract] OR viscoelastic[Title/Abstract] OR thromboelast*[Title/Abstract]
OR ROTEM[Title/Abstract] OR TEG[Title/Abstract] OR VET[Title/Abstract]) AND ("2020/10/23"[Date - Publication] :
"2026/10/23"[Date - Publication])
```

The two queries retrieved 147 records in total, representing 133 unique PMIDs after 14 overlapping records were removed. These counts describe only these two documented searches. They do not reconstruct the original searches in PubMed/MEDLINE, Scopus or Web of Science, and do not constitute a systematic-review flow or the number of studies included.

The documented searches supported title-level assessment and targeted abstract/full-text verification of bleeding definitions, direct FET evidence, haemostatic management, and device coverage. No exhaustive title/abstract eligibility assessment or duplicate independent screening is claimed. All authors contributed to literature identification, selection, critical appraisal and interpretation. Older landmark sources were retained for definitions and mechanistic context.

The six SANRA domains were considered while revising importance, aims, search description, referencing, scientific reasoning and endpoint presentation. No numerical SANRA rating, inter-rater agreement or formal risk-of-bias assessment is reported.

Table S2 Cited evidence and its role in the narrative synthesis

All 41 cited publications are listed below. “Tabulated FET clinical publication” refers to 12 primary clinical publications represented in the main tables; it excludes the pooled review and the case-based technical report. It is a count of publications, not independent cohorts or a non-overlapping patient total.

Ref.	Publication	Evidence role and use
1	Tsagakis, K.; Pacini, D.; Grabenwöger, M.; Borger, M.A.; Goebel, N.; Hemmer, W.; Santos, A.L.; Sioris, T.; Widenka, K.; Risteski, P.; et al. Results of frozen elephant trunk from the international E-vita Open registry. Ann. Cardiothorac. Surg. 2020, 9, 178-188. https://doi.org/10.21037/acs-2020-fet-25.	Tabulated FET clinical publication. International registry; postoperative re-exploration and procedural context.

Ref.	Publication	Evidence role and use
2	Shrestha, M.; Bachet, J.; Bavaria, J.; Carrel, T.P.; De Paulis, R.; Di Bartolomeo, R.; Etz, C.D.; Grabenwöger, M.; Grimm, M.; Haverich, A.; et al. Current status and recommendations for use of the frozen elephant trunk technique: a position paper by the Vascular Domain of EACTS. Eur. J. Cardiothorac. Surg. 2015, 47, 759-769. https://doi.org/10.1093/ejcts/ezv085.	Position paper. FET indications and procedural context.
3	Di Bartolomeo, R.; Di Marco, L.; Armaro, A.; Marsilli, D.; Leone, A.; Pilato, E.; Pacini, D. Treatment of complex disease of the thoracic aorta: the frozen elephant trunk technique with the E-vita open prosthesis. Eur. J. Cardiothorac. Surg. 2009, 35, 671-676. https://doi.org/10.1016/j.ejcts.2008.12.010.	FET background clinical evidence. Early E-vita technique and clinical experience; not a harmonised haemostatic dataset.
4	Leontyev, S.; Tsagakis, K.; Pacini, D.; Di Bartolomeo, R.; Mohr, F.W.; Weiss, G.; Grabenwoeger, M.; Mascaro, J.G.; Iafrancesco, M.; Franke, U.F.; et al. Impact of clinical factors and surgical techniques on early outcome of patients treated with frozen elephant trunk technique by using EVITA open stent-graft: results of a multicentre study. Eur. J. Cardiothorac. Surg. 2016, 49, 660-666. https://doi.org/10.1093/ejcts/ezv150.	FET background clinical evidence. Clinical/procedural factors in multicentre FET experience.
5	Kayali, F.; Chikhal, R.; Agbobu, T.; Jubouri, M.; Patel, R.; Chen, E.P.; Mohammed, I.; Bashir, M. Evidence-based frozen elephant trunk practice: a narrative review. Cardiovasc. Diagn. Ther. 2023, 13, 1104-1117. https://doi.org/10.21037/cdt-23-300.	FET narrative review. Procedure and evidence context.
6	Bashir, M.; Abo Aljadayel, H.; Mousavizadeh, M.; Daliri, M.; Rezaei, Y.; Tan, S.Z.; Mohammed, I.; Hosseini, S. Correlation of coagulopathy and frozen elephant trunk use in aortic arch surgery: A systematic review and meta-analysis. J. Card. Surg. 2021, 36, 4699-4714. https://doi.org/10.1111/jocs.16001.	FET systematic review. Pooled endpoints; heterogeneous definitions; not an independent clinical cohort.
7	Agarwal, S.; Abdelmotieleb, M. Viscoelastic testing in cardiac surgery. Transfusion 2020, 60, S52-S60. https://doi.org/10.1111/trf.16075.	Contextual narrative review. Viscoelastic functional assessment in cardiac surgery.
8	Dyke, C.; Aronson, S.; Dietrich, W.; Hofmann, A.; Karkouti, K.; Levi, M.; Murphy, G.J.; Sellke, F.W.; Shore-Lesserson, L.; von Heymann, C.; et al. Universal definition of perioperative bleeding in adult cardiac surgery. J. Thorac. Cardiovasc. Surg. 2014, 147, 1458-1463.e1. https://doi.org/10.1016/j.jtcvs.2013.10.070.	Consensus definition. UDPB severity reporting and original component rules.
9	Biancari, F.; Ruggieri, V.G.; Perrotti, A.; Svenarud, P.; Dalén, M.; Onorati, F.; Faggian, G.; Santarpino, G.; Maselli, D.; Dominici, C.; et al. European Multicenter Study on Coronary Artery Bypass Grafting (E-CABG registry): Study Protocol for a Prospective Clinical Registry and Proposal of Classification of Postoperative Complications. J. Cardiothorac. Surg. 2015, 10, 90. https://doi.org/10.1186/s13019-015-0292-z.	Registry protocol/classification proposal. E-CABG framework, originally in CABG.
10	Mehran, R.; Rao, S.V.; Bhatt, D.L.; Gibson, C.M.; Caixeta, A.; Eikelboom, J.; Kaul, S.; Wiviott, S.D.; Menon, V.; Nikolsky, E.; et al. Standardized bleeding definitions for cardiovascular clinical trials: a consensus report from the Bleeding Academic Research Consortium. Circulation 2011, 123, 2736-2747. https://doi.org/10.1161/circulationaha.110.009449.	Consensus definition. BARC definitions, including CABG-related category.
11	El-Sayed Ahmad, A.; Silaschi, M.; Borger, M.; Seidiramool, V.; Hamiko, M.; Leontyev, S.; Zierer, A.; Doss, M.; Etz, C.D.; Benedikt, P.; et al. The Frozen Elephant Technique Using a Novel Hybrid Prosthesis for Extensive Aortic Arch Disease: A Multicentre Study. Adv. Ther. 2023, 40, 1104-1113. https://doi.org/10.1007/s12325-022-02418-5.	Tabulated FET clinical publication. Multicentre clinical series; drainage and 24-h treatment burden.
12	Kim, C.H.; Kim, T.-H.; Lee, H.; Kim, M.S.; Heo, W.; Yoo, K.-J.; Cho, B.-K.; Song, S.-W. One-year outcomes of total arch replacement and frozen elephant trunk using the E-vita Open NEO. Eur. J. Cardiothorac. Surg. 2024, 65, ezae017. https://doi.org/10.1093/ejcts/ezae017.	Tabulated FET clinical publication. Clinical series; multiple drainage windows and re-exploration.

Ref.	Publication	Evidence role and use
13	Tsagakis, K.; Kempfert, J.; Zierer, A.; Martens, A.; Dohle, D.-S.; Castiglioni, A.; Wong, R.H.-L.; Widenka, K.; Liakopoulos, O.; Borger, M.A.; et al. E-vita OPEN NEO in the treatment of acute or chronic aortic pathologies: first interim results of the NEOS study. Eur. J. Cardiothorac. Surg. 2024, 65, ezae206. https://doi.org/10.1093/ejcts/ezae206.	Tabulated FET clinical publication. Prospective multicentre interim study; graft observations and early re-exploration.
14	Demal, T.J.; Bax, L.; Brickwedel, J.; Kölbel, T.; Vettorazzi, E.; Sitzmann, F.; Reichenspurner, H.; Detter, C. Outcome of the frozen elephant trunk procedure as a redo operation. Interact. Cardiovasc. Thorac. Surg. 2021, 33, 85-92. https://doi.org/10.1093/icvts/ivab059.	Tabulated FET clinical publication. Primary versus redo clinical cohort.
15	Kozlov, B.; Panfilov, D.; Lukinov, V. Frozen Elephant Trunk for Aortic Dissection Using Different Hybrid Grafts: Preliminary Results from a Prospective Study. J. Pers. Med. 2023, 13, 784. https://doi.org/10.3390/jpm13050784.	Tabulated FET clinical publication. Prospective comparison of hybrid grafts; postoperative endpoints.
16	Xie, E.; Wu, J.; Qiu, J.; Dai, L.; Qiu, J.; Luo, Q.; Jiang, W.; Cao, F.; Zhao, R.; Fan, S.; et al. Early Outcomes of Three Total Arch Replacement Strategies for DeBakey Type I Aortic Dissection. Front. Cardiovasc. Med. 2021, 8, 638420. https://doi.org/10.3389/fcvm.2021.638420.	Tabulated FET clinical publication. Three-strategy clinical comparison; weighted and unweighted results distinguished.
17	Ho, J.Y.K.; Lim, K.; Fujikawa, T.; Wong, R.H.L. Pre-emptive BioGlue priming of the branched Evita Open NEO hybrid prosthesis: tackling excessive oozing. Eur. J. Cardiothorac. Surg. 2022, 61, 728-729. https://doi.org/10.1093/ejcts/ezab398.	FET technical report. Case-based graft priming and haemostatic experience.
18	Czerny, M.; Beyersdorf, F.; Murana, G.; Pacini, D. Excessive oozing through the fabric of the branched Cryolife-Jotec Evita Open NEO hybrid prosthesis. Eur. J. Cardiothorac. Surg. 2021, 60, 423-424. https://doi.org/10.1093/ejcts/ezab058.	FET clinical technical communication. Clinical fabric-oozing observations; limited generalisability.
19	Tan, S.Z.C.P.; Bashir, M. Prevention versus cure: Is BioGlue priming the optimal strategy against E-Vita NEO graft oozing?. J. Card. Surg. 2022, 37, 555-560. https://doi.org/10.1111/jocs.16179.	Experimental/technical evidence. Perfused graft model; permeability and BioGlue concerns.
20	Jakob, H.; Leins, T.; Avci-Adali, M.; Schlensak, C.; Wendt, D.; Mehta, Y.; Thielmann, M.; Görlinger, K.; Song, S.-W.; Tsagakis, K. Standardized in vitro bleeding tests in a non-coated novel hybrid prosthesis for frozen elephant trunk demonstrates minimal oozing during full heparinization, supported by clinical data. Front. Cardiovasc. Med. 2023, 10, 1303816. https://doi.org/10.3389/fcvm.2023.1303816.	Tabulated FET clinical publication. Mixed experimental/clinical report; Essen and Seoul cohorts kept separate.
21	Zindovic, I.; Sjögren, J.; Bjursten, H.; Ingemansson, R.; Ingimarsson, J.; Larsson, M.; Svensson, P.J.; Strandberg, K.; Wierup, P.; Nozohoor, S. The Coagulopathy of Acute Type A Aortic Dissection: A Prospective, Observational Study. J. Cardiothorac. Vasc. Anesth. 2019, 33, 2746-2754. https://doi.org/10.1053/j.jvca.2019.02.013.	Contextual acute dissection study. Prospective haemostatic abnormalities; not exclusively FET.
22	Arima, D.; Suematsu, Y.; Yamada, R.; Matsumoto, R.; Kurahashi, K.; Nishi, S.; Yoshimoto, A. Relationship of acute type A aortic dissection and disseminated intravascular coagulation. J. Vasc. Surg. 2022, 75, 1553-1560.e1. https://doi.org/10.1016/j.jvs.2021.12.064.	Contextual acute dissection evidence. Disseminated coagulation abnormalities.
23	Gao, Z.; Pei, X.; He, C.; Wang, Y.; Lu, J.; Jin, M.; Cheng, W. Oxygenation impairment in patients with acute aortic dissection is associated with disorders of coagulation and fibrinolysis: a prospective observational study. J. Thorac. Dis. 2019, 11, 1190-1201. https://doi.org/10.21037/jtd.2019.04.32.	Contextual acute dissection study. Coagulation/fibrinolysis and oxygenation.
24	Jakob, H.; Idhrees, M.; Bashir, M. From E-VITA open plus to E-VITA NEO and E-NOVIA. J. Card. Surg. 2021, 36, 1814-1817. https://doi.org/10.1111/jocs.15044.	Device background. Evolution and description of hybrid devices.
25	Rorris, F.-P.; Antonopoulos, C.N.; Gissis, I.; Tsagakis, K.; Kokotsakis, J. E-Vita OPEN NEO Hybrid Stent Graft: A New Frontier for Total Arch Replacement. Ann. Vasc. Surg. 2022, 84, 211-217. https://doi.org/10.1016/j.avsg.2022.01.034.	FET initial clinical/device report. Six-patient experience and device/technique; not direct support for fabric oozing in the abstract.

Ref.	Publication	Evidence role and use
26	Baethge, C.; Goldbeck-Wood, S.; Mertens, S. SANRA—a scale for the quality assessment of narrative review articles. Res. Integr. Peer Rev. 2019, 4, 5. https://doi.org/10.1186/s41073-019-0064-8.	Methodological instrument. SANRA critical appraisal of narrative reviews.
27	Casselman, F.P.A.; Lance, M.D.; Ahmed, A.; Ascari, A.; Blanco-Morillo, J.; Bolliger, D.; Eid, M.; Erdoes, G.; Haumann, R.G.; Jeppsson, A.; et al. 2024 EACTS/EACTAIC Guidelines on patient blood management in adult cardiac surgery in collaboration with EBCP. Eur. J. Cardiothorac. Surg. 2025, 67, ezae352. https://doi.org/10.1093/ejcts/ezae352.	Professional guideline. Adult cardiac-surgery patient blood management; not FET validation.
28	Abbasciano, R.G.; Koulouroudias, M.; Chad, T.; Mohamed, W.; Leeman, I.; Pellowe, C.; Kunst, G.; Klein, A.; Murphy, G.J. Role of Hypothermia in Adult Cardiac Surgery Patients: A Systematic Review and Meta-analysis. J. Cardiothorac. Vasc. Anesth. 2022, 36, 1883-1890. https://doi.org/10.1053/j.jvca.2022.01.026.	Contextual systematic review. Hypothermia in adult cardiac surgery.
29	Xiao, F.-C.; Ma, W.-G.; Ge, Y.-P.; Zhu, J.-M.; Sun, L.-Z. Does preoperative dual antiplatelet therapy affect bleeding and mortality after total arch repair for acute type A dissection?. Interact. Cardiovasc. Thorac. Surg. 2022, 34, 120-127. https://doi.org/10.1093/icvts/ivab226.	Tabulated FET clinical publication. Preoperative DAPT; explicit 12-h component and composite endpoint.
30	Vincentelli, A.; Susen, S.; Le Tourneau, T.; Six, I.; Fabre, O.; Juthier, F.; Bauters, A.; Decoene, C.; Goudemand, J.; Prat, A.; et al. Acquired von Willebrand Syndrome in Aortic Stenosis. N. Engl. J. Med. 2003, 349, 343-349. https://doi.org/10.1056/nejmoa022831.	Contextual aortic stenosis study. Acquired von Willebrand abnormalities with severe stenosis.
31	Pfeiffer, P.; Buchholz, V.; Probst, C.; Ghazy, A.; Rissel, R.; Griesinger, J.; Treede, H.; Dohle, D.-S. Transfusion and coagulation management in acute type A aortic dissection. Arch. Cardiovasc. Dis. 2025, 118, 365-373. https://doi.org/10.1016/j.acvd.2025.02.004.	Contextual acute dissection management evidence. Transfusion/coagulation context; avoid treating as FET-specific validation.
32	Monaco, F.; Nardelli, P.; Denaro, G.; De Luca, M.; Franco, A.; Bertoglio, L.; Castiglioni, A.; Zangrillo, A. First experience with a ROTEM-enhanced transfusion algorithm in patients undergoing aortic arch replacement with frozen elephant trunk technique. A theranostic approach to patient blood management. J. Clin. Anesth. 2020, 66, 109910. https://doi.org/10.1016/j.jclinane.2020.109910.	Tabulated FET clinical publication. Retrospective before/after management protocol; bundled intervention.
33	Tibi, P.; McClure, R.S.; Huang, J.; Baker, R.A.; Fitzgerald, D.; Mazer, C.D.; Stone, M.; Chu, D.; Stammers, A.H.; Dickinson, T.; et al. STS/SCA/AmSECT/SABM Update to the Clinical Practice Guidelines on Patient Blood Management. Ann. Thorac. Surg. 2021, 112, 981-1004. https://doi.org/10.1016/j.athoracsur.2021.03.033.	Professional guideline. Cardiac-surgery patient blood management, antifibrinolytics and salvage.
34	Bojko, M.M.; Oslund, W.; Kirsch, M.J.; Carroll, A.M.; Longo, E.; Clothier, J.S.; Okonkwo, K.; Rajeev, N.; Dhanekula, A.; McCarthy, F.; et al. Commercial hybrid graft versus traditional arch replacement with frozen elephant trunk: A multi-institutional comparison. JTCVS Open 2025, 23, 19-33. https://doi.org/10.1016/j.xjon.2024.10.027.	Tabulated FET clinical publication. Thoraflex versus traditional FET; matched multicentre comparison.
35	Hanke, A.A.; Herold, U.; Dirkmann, D.; Tsagakis, K.; Jakob, H.; Görlinger, K. Thromboelastometry Based Early Goal-Directed Coagulation Management Reduces Blood Transfusion Requirements, Adverse Events, and Costs in Acute Type A Aortic Dissection: A Pilot Study. Transfus. Med. Hemother. 2012, 39, 121-128. https://doi.org/10.1159/000337723.	Contextual acute dissection pilot. Goal-directed coagulation management; no independent FET validation.
36	Shore-Lesserson, L.; Manspeizer, H.E.; DePerio, M.; Francis, S.; Vela-Cantos, F.; Ergin, M.A. Thromboelastography-Guided Transfusion Algorithm Reduces Transfusions in Complex Cardiac Surgery. Anesth. Analg. 1999, 88, 312-319. https://doi.org/10.1097/00000539-199902000-00016.	Contextual complex cardiac study. TEG-guided transfusion management.
37	Haensig, M.; Kempfert, J.; Kempfert, P.-M.; Girdauskas, E.; Borger, M.A.; Lehmann, S. Thrombelastometry guided blood-component therapy after cardiac surgery: a randomized study. BMC Anesthesiol. 2019, 19, 201. https://doi.org/10.1186/s12871-019-0875-7.	Contextual cardiac randomised study. Thromboelastometry-guided blood components.

Ref.	Publication	Evidence role and use
38	Torre, D.E.; Pirri, C. Aortic Arch and Frozen Elephant Trunk Surgery: Anesthetic Challenges and Strategies for Organ Protection. J. Clin. Med. 2026, 15, 877. https://doi.org/10.3390/jcm15020877.	FET/arch narrative review. Anaesthetic strategy context, not prospective trigger validation.
39	Doll, I.; Salewski, C.; Vöhringer, L.; Baumgaertner, M.; Nemeth, A.; Schlensak, C.; Radwan, M. Early and 3-Year Outcomes of Frozen Elephant Trunk Procedure with Evolving E-vita Hybrid Grafts: A Retrospective Single-Centre Cohort Study over 11 Years. J. Cardiovasc. Dev. Dis. 2025, 12, 368. https://doi.org/10.3390/jcdd12090368.	Tabulated FET clinical publication. Single-centre cohort; treatment exposure and clinical outcomes.
40	Guan, X.; Li, L.; Lu, X.; Gong, M.; Li, H.; Liu, Y.; Jiang, W.; Lan, F.; Wang, X.; Zhang, H. Safety and efficacy of fibrinogen concentrate in aortic arch surgery involving moderate hypothermic circulatory arrest. J. Thromb. Thrombolysis 2023, 55, 67-73. https://doi.org/10.1007/s11239-022-02706-5.	Contextual arch observational study. Low fibrinogen and replacement during moderate HCA.
41	Vlot, E.A.; Hackeng, C.M.; Aper, S.J.A.; Sonker, U.; Heijmen, R.H.; van Dongen, E.P.A.; Noordzij, P.G. Does Intraoperative Fibrinogen Affect Blood Loss or Transfusion Practice After Aortic Arch Surgery: A Prematurely Ended Randomized Trial. Clin. Appl. Thromb. Hemost. 2022, 28, 10760296221144042. https://doi.org/10.1177/10760296221144042.	Contextual arch randomised study. Fibrinogen/placebo under deep HCA; prematurely stopped.

Abbreviations: BARC, Bleeding Academic Research Consortium; CABG, coronary artery bypass grafting; DAPT, dual antiplatelet therapy; E-CABG, European Multicenter Study on CABG; FET, frozen elephant trunk; HCA, hypothermic circulatory arrest; ROTEM, rotational thromboelastometry; SANRA, Scale for the Assessment of Narrative Review Articles; TEG, thromboelastography; UDPB, Universal Definition of Perioperative Bleeding.

Supplementary Table S3
Supplementary Study Level Reporting Map

Manuscript jcm-4637756

Table S3. Study-level reporting map for the clinical publications in Tables 1 and 2 of the revised manuscript. Reference numbers refer to the main manuscript.

Domains correspond to Table 3 and Figure 2: A, procedural context; B, bleeding measurement; C, haemostatic status; D, verified bleeding source; E, treatment exposure; F, outcomes and severity reporting.

Clinical publication	A	B	C	D	E	F
Tsagakis registry [1]	R	U	U	U	U	R
Demal [14]	R	P	U	U	P	R
Xie [16]	R	P	U	U	P	R
Kozlov [15]	R	P	U	U	P	R
El-Sayed Ahmad [11]	R	P	U	P	R	R
Jakob [20]	R	P	U	P	P	R
Kim [12]	R	P	U	U	P	R
NEOS [13]	R	U	U	P	U	R

Clinical publication	A	B	C	D	E	F
Xiao [29]	R	P	U	U	P	R
Bojko [34]	R	U	U	U	R	R
Monaco [32]	R	P	R	U	R	R
Doll [39]	R	U	P	U	P	R

R, relevant information is extractable for that domain, not necessarily every proposed field; P, only partial or qualitative information is extractable; U, the domain cannot be established from the haemostatic dataset extracted for this review. U is not proof that a test or observation was not performed. This descriptive map does not assign individual-patient sources, reconstruct UDPB from cohort averages or measure reporting quality numerically. Laboratory, timing and source fields remain particularly incomplete. UDPB, Universal Definition of Perioperative Bleeding.

The map describes the information extracted for this narrative review, rather than a formal reporting-quality score. R does not imply complete reporting of every proposed variable. P and U do not establish that a clinical assessment or intervention was omitted. Source findings cannot be reconstructed from aggregate drainage, transfusion or re-exploration rates.
